# Escala de evaluación de artículos con metodologías heterogéneas para revisiones integrativas*

**DOI:** 10.15649/cuidarte.2744

**Published:** 2022-10-22

**Authors:** Miguel Andrez Valencia-Contrera

**Affiliations:** 1 . Universidad de Antofagasta, Antofagasta, Chile. Estudiante de Doctorado en Ciencia de Enfermería, Universidad Andrés Bello, Santiago, Chile. Email: miguel.valencia@uantof.cl Universidad de Antofagasta Universidad de Antofagasta Antofagasta Chile miguel.valencia@uantof.cl

## Abstract

Recientemente ha sido publicada la escala de Evaluación de Artículos con Metodologías Heterogéneas para Revisiones Integrativas (EAMH)^1^, esta se generó en respuesta a la creciente necesidad de asegurar la calidad de los resultados en revisiones integrativas, la propuesta proporciona orientaciones sobre criterios básicos que cualquier artículo debe cumplir para ser incluidos en el análisis de los resultados. La escala consta de 6 preguntas evaluadoras, cuyas respuestas son dicotómicas “SI/NO”, cada respuesta “SI” presenta un punto, por lo tanto, su puntaje varía entre cero a seis puntos, y su interpretación es la siguiente: de 0 a 3 puntos “artículo no recomendable para el análisis”; de 4 a 5 puntos “artículo apto para el análisis”; y finalmente, 6 puntos “artículo ideal para el análisis”. A continuación, se expondrá los resultados de la aplicación de dicha escala en el análisis de artículos publicados en la presente revista, periodo 2021-2022 hasta el mes de abril (ver Tabla 1). La base de datos fue almacenada en Mendeley Data^2^.

Tras el análisis, el 13,4%(n=30) de los artículos usados como muestra en las revisiones analizadas^3^^-^^11^, obtuvieron de 0 a 3 puntos, clasificación correspondiente a “artículo no recomendable para el análisis”, ya que no lograron cumplir con la mayoría de las preguntas evaluadoras contenidas en la escala. Cabe destacar que del total de artículos usados como muestra, ninguno utilizó criterios de calidad en sus respectivas muestras, más bien se utilizaron: clasificaciones de nivel de evidencia; preguntas clínicas; o aplicación de Plantillas del Critical Appraisal Skills Programme en español (CASPe).

**Tabla 1:** Análisis según la escala EAMH de revisiones integrativas publicadas en la revista cuidarte periodo 2021-2022

Artículos analizados	Total de citas utilizadas como muestra	Artículos utilizados como muestra y clasificados según categorías de la escala EAMH	Investigaciones no recuperadas
		0-3 puntos 4-5 puntos 6 puntos	
_9_^3^^-^^11^	223	30 18 165	10

Los resultados dan indicios que la aplicación de la escala en los artículos evaluados podría mejorar la calidad de la muestra en revisiones integrativas, considerado por el presente autor como una propuesta que contribuye al logro de la rigurosidad pletórica que ha de tener un investigador en su escrutinio. El presente documento no ha de tener una intención de criticar o cuestionar el trabajo de otros colegas, más aún cuando superaron una etapa de revisión, sino más bien, tributar hacia la acuciosidad que el proceso amerita, relevando la importancia de analizar la calidad de los artículos seleccionados en una revisión.

El que un artículo haya cumplido con los criterios de inclusión y exclusión definidos por un investigador, no significa que sea ideal para el análisis de los resultados, a no ser que considere la calidad de estos, puesto que muchos de ellos pueden contener falencias en la presentación de los objetivos, metodologías, discordancias entre objetivo y metodología, ausencia de justificación de la cantidad y tipo de la muestra, ausencia de información que dé cuenta cómo se accedió a la muestra, y los resultados o conclusiones pueden no responden al objetivo planteado; en dicho escenario, incurrir en este error, podría conllevar el generar un resultado sesgado en una revisión, a su vez, este puede ser utilizado por la comunidad científica y ser replicado, generando un círculo vicioso que atenta al rigor científico, es ahí donde se enmarca la necesidad de utilizar medios que subsanen aquellos elementos que merman la calidad de los resultados.

## References

[1] Valencia-Contrera MA, Orellana-Yañez AE (2022). Fenómeno techo de cristal en enfermería: revisión integrativa. Rev Cuid..

[2] Valencia-Contrera MA (2022). Escala de evaluación de artículos con metodologías heterogéneas para revisiones integrativas.

[3] Barros FRB de, Lima RF da S, Magalhaes VM de P (2021). Tecnologías desenvolvidas no contexto da saúde da mulher no Brasil: uma revisao integrativa. Rev Cuid..

[4] Ardila Suárez EF, Arredondo Holguín E del S (2021). Actividades de enfermería para la satisfacción de necesidades familiares en cuidado intensivo adulto: una revisión integrativa. Rev Cuid..

[5] Jesus SC, Huller Farias C, Schneider DG, Schoeller SD, Bertoncello KCG (2021). Honneth: Contributes para o cuidar em enfermagem a luz do amor, direito e solidariedade. RevCuid..

[6] Guáqueta Parada SR, Henao-Castaño AM, Motta Robayo CL, Triana Restrepo MC, Burgos Herrera JD, Neira Fernández KD, Peña Almanza BA (2021). Intervenciones de Enfermería ante la Necesidad de Información de la Familia del Paciente Crítico. Rev Cuid..

[7] Fernandes C da S, Brandao MGSA, Lima MM de S, Nascimento JC do, Neto NMG, Barros LM (2021). Práticas seguras no manejo de vias aéreas de pacientes con Covid-19: revisión integradora. Rev Cuid..

[8] Araújo Rocha G, Oliveira AKL de, Oliveira FGL, Rodrigues VES, Moura AG de S, Sousa EB, Machado ALG (2021). Cuidados com o acesso vascular para hemodiálise: revisao integrativa. Rev Cuid..

[9] Gómez Barriga N, Medina Garzón M (2021). Intervenciones de Enfermería en la reversión del estoma intestinal: revisión integrativa. Rev Cuid..

[10] Fiorentin L, Beltrame V (2022). Distanciamento social por Covid 19: repercussao na rotina de universitários. RevCuid..

[12] Castiblanco Montañez RA, Coronado Veloza CM, Morales Ballesteros LV, Polo González TV, Saavedra Leyva AJ (2022). Hemorragia postparto: intervenciones y tratamiento del profesional de enfermería para prevenir shock hipovolémico. Rev Cuid..

